# Pain Relief in Dental Local Anaesthesia with Vibrational Devices: Much Ado about Nothing? A Scoping Review

**DOI:** 10.3390/jcm12041448

**Published:** 2023-02-11

**Authors:** Alessandra Putrino, Maria Rosaria Abed, Enrico Marinelli, Simona Zaami

**Affiliations:** 1 Department of Anatomical, Histological, Forensic and Orthopedic Sciences, Sapienza University of Rome, 00161 Rome, Italy; 2 Independent Researcher, 28045 Madrid, Spain; 3 Department of Medico-Surgical Sciences and Biotechnologies, Sapienza University of Rome, 04100 Latina, Italy

**Keywords:** pain, anxiety, anaesthesia, dentistry, paediatric dentistry, orthodontics, quality of life, mouth rehabilitation, vibration

## Abstract

In recent years, vibrational devices have been introduced in order to reduce patient discomfort in some situations such as orofacial pain, orthodontic therapy, and injection of local anaesthetics. This article aims to review the clinical experience given by the use of these devices in local anaesthesia. The literature search was carried out on the main scientific databases for articles up to November 2022. Eligibility criteria were established, and pertinent articles selected. The results were classified by author, year, type of study, sample size and characteristics, purpose of use, type of vibrational device used, protocol used, and outcomes. Nine relevant articles were found. These are split mouth randomized clinical trials which evaluate the reduction in pain perception with different devices and different protocols of use in children, during procedures which require local analgesia by injection, compared with traditional local anaesthesia with premedication based on anaesthetic gels. Different objective and subjective scales of pain and discomfort perception were used. Although results are promising, some data, such as those relating to vibrational intensity and frequency, are not clear. Evaluations on samples varying by age and context of use are necessary to fully define the indications for this type of aid during oral rehabilitation procedures.

## 1. Introduction 

According to the International Association for the Study of Pain “Pain is an unpleasant sensory and emotional experience associated with defined or potential tissue damage or described in respect to such damage” [1]. The fear of dental procedures and treatments is one of the strongest fears in both paediatric and adult age groups [2,3,4]. This is particularly related to dental procedures in which needles are used, such as in local anaesthesia [5,6,7]. This happens mainly because patients often start dental sessions due to the presence of painful lesions (caries, periapical lesions, dental fractures, etc.), so are in a condition of stress and generalized fear [8,9,10]. The functional limitation due to temporomandibular dysfunction is often already accompanied by symptoms such as pain, and its therapeutic approach can itself stimulate the perception of pain and discomfort [11,12,13,14]. Even orthodontic therapy, regardless of the type of device used, is often perceived by patients as a painful and unpleasant [15,16,17,18,19]. For a long time, great attention has been paid to the possibility that alternative methods to the oral administration of analgesics, properly conveyed, can reduce the perception of various types of dental pain by promoting a state of well-being in the patient [20]. Transcutaneous electrical nerve stimulation (TENS), acupuncture, vibration, and conditioned pain modulation (CPM) are methods capable of controlling mechanisms underlying pain inhibition [20,21]. Much has been written on these topics and research continues to be carried out in this field [22,23]. In particular, the application of vibrational stimuli of low (30 Hz) or higher (up to 120 Hz) intensity would be able to reduce both the sensation of orthodontic dental pain, for a stimulation of local cellular metabolism that includes modulation of periodontal chemical mediators of inflammation (also involved in orthodontic movement due to an effect on bone remodelling), and orofacial and temporo-mandibular joint pain for a more extensive action that, similarly, improves blood and lymphatic circulation by relieving pain and improving functional recovery [24,25,26,27]. Local anaesthesia is one of the most frequently needed procedures to rehabilitate the patient, and is also the one often perceived as most painful and undesirable [6,7,8,28]. Many recent studies have documented the use of additional vibrational stimuli to relieve pain during the administration of local anaesthesia [29,30,31]. Yet, there are still no reviews of the literature on the subject. The main objective of this scoping review is to focus on the differences between any existing protocols (applied strength and frequency) and available devices to determine whether vibrations can significantly contribute to reducing pain in the administration of local anaesthesia during dental procedures.

## 2. Materials and Methods

**Figure 1:**
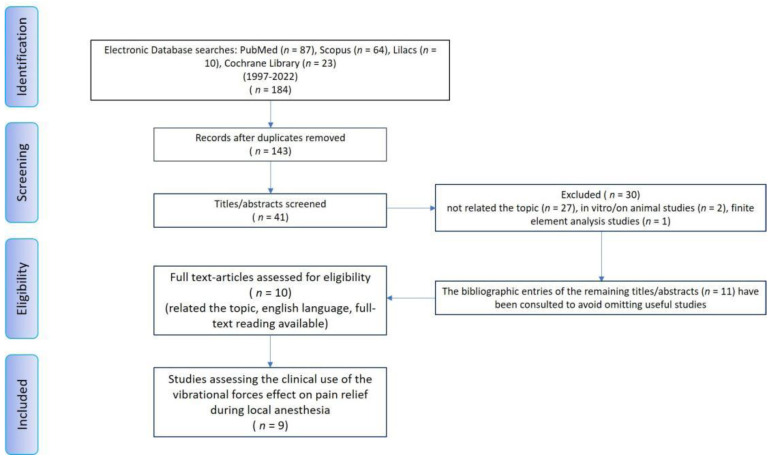
Review flow diagram for the scoping review detailing the database searches, the number of abstract screened, and the full texts retrieved.

This scoping review project has been now (27 December 2022) registered as an Open-Ended Registration on OSF Registries (Open Science Framework, Center for Open Science©, 2011–2022). The search for sources for this scoping review of the literature began on 28 November 2022 and ended on 30 November 2022 by consulting four databases: PubMed, Scopus, Lilacs, and the Cochrane Library (Figure 1). Based on the acronym PCC (Population/Problem, Concept, Context), the research question that was set was the following: “Do dental patients under the effect of additional vibrational forces have a pain relief during dental local anaesthesia?” (Table 1). The search was conducted using the following MeSH terms and free terms in combination with the Boolean operators “AND” and “OR”: vibration, dentistry, paediatric dentistry, mouth, anaesthesia, pain. The two operators involved in the research of the sources carried out their activity independently, respecting specific inclusion criteria. The studies included in the review were of the randomized and non-randomized comparative clinical type. In vitro studies, finite element analysis studies, animal studies, case reports (and case series), and reviews were not eligible (Table 2). The bibliographic entries of each selected study and any previous reviews, when available, were consulted to avoid leaving out studies useful for this scoping review that may not have been among the search results. There were no restrictions on the year of publication. Only studies in English, for which abstract and full text were available, were ultimately considered. The results of the source search conducted by each operator were collected separately and then merged by Zotero software (Zotero 5.0 for Windows, Corporation for Digital Scholarship, Vienna, VA, USA) to eliminate any duplicates. In case of doubts about the contents of the abstracts of some studies, these were included for the full text reading and subsequent evaluation. Both review practitioners subsequently read the selected studies individually and confirmed or rejected inclusion in the scoping review. In the event of conflicting opinions, they consulted each other until they reached an unambiguous opinion. The two reviewers independently charted the data and discussed the results. Data related to each study characteristic included in the review were abstracted. In the results section, the studies will be grouped and summarized by study design, types of devices, and protocols used, only if appropriate.

## 3. Results

The database search initially yielded 184 results (PubMed 87, Lilacs 10, Cochrane Library 23, Scopus 64). Duplicates were removed with the help of the Zotero software. Following this phase, the remaining 41 results were checked for eligibility criteria by reading the abstracts. With this selection, 30 scientific articles were further discarded because they did not meet the eligibility criteria. Of the remaining 11 articles, useful for the review, the bibliographic entries were consulted to look for any further studies suitable for the review that had not emerged from the initial search. No other studies resulted from this supplemental research; so, only the original 10 articles were confirmed for the scoping review by full text reading. One study has been excluded because the full-text article was not available also after request to the authors, and another one was excluded because no outcome was reported. Therefore, only nine articles were included in the scoping review (Figure 1). The included studies were classified by author, year, type of study, sample size and its characteristics (sex, age), goal of the research, type of vibrational device used, protocol used, and pain relief as the outcome (Table 3).

The studies subject to the scoping review were published between 1997 and 2022. The total number of patients involved in studies on the use of supplementary vibrations during local anaesthesia in dental procedures should be equal to 611 subjects. Considering that only three studies specify the sex of the patients involved, we cannot state the distribution between the two sexes is homogeneous. Only three studies do not clarify the range of age of the patients [29,32,33]; overall, the patients involved are aged between 5 and 12 years, except for the two studies which do not clarify age range and classify their patients as adults [32,33]. Regarding the type of study, all the studies were split mouth randomized clinical trials. The experimental approach always included two sessions: one in which the traditional procedure is used in a dental hemiarch (premedication with anaesthetic gel and subsequent infiltration of the anaesthetic with syringe), and the other in which the vibrational device is applied before and during the injection. The experimental and control sides as well as which of the two interventions to perform in the first and second sessions are chosen randomly. On the control side, premedication with anaesthetic gels is carried out with 20% benzocaine [29,32,38], 8% lignocaine and 0.8% dibucaine [34], lignocaine hydrochloride 2% [31,33,36]. Injections infiltrate lidocaine with 1:100,000 epinephrine in all the studies except one where dental analgesia is obtained with Ubistesin-4% Articaine with epinephrine 1:200,000. Needles, when specified, are all around 24–27 gauges. Indications to dental anaesthesia in patients recruited in the research are not explained. Sites for injections, when the information is specified, are palatal [33] infraorbital [33], upper posterior buccal [32,33,37], inferior alveolar nerve block [33,36,37], and posterior palatal [37]. The vibrational devices used are a modified battery-powered shaver (Windmere Corp., Miami, FL) [32], DentalVibe^®^ Injection Comfort system (BING Innovations, FL, USA) [29,33,34,36], Buzzy^®^ (MMJ Labs, Atlanta GA, USA) [31,35], and Vibraject (Vibraject^®^MiltexInc LLC., York, PA, USA) [37]. One study shows the photo of the device used but does not give a trade name or provide information about its origin [30]. Except for one study, the others do not clarify what frequency or vibrational intensity the device is set to [32]. In all studies, the vibrational device is applied before, during, and after the injection of anaesthetic, but it is not always clear how long before and after the injection it is activated, and if there are any precautions to consider for better use. In the two studies where the Buzzy^®^ device is used [31,35], a gel ice pack comprising water, sodium polyacrylate, and mixed isothiazolinones cooled to 5 °C for 30 s is applied to improve pain relief with vibrational stimulation. The response of the subjects involved in the trials is evaluated according to objective or subjective scales of measurement of stress, discomfort, and pain. In addition to heart rate and pO_2_ saturation [30,35,36], the most used scales were Iowa Cancer Pain Relief Initiative scale [32], VAS (visual analogue scale) [33,37], Wong–Baker score pain scale [35,36], FLACC (Faces, Legs, Activity, Cry, Consolability) score [30,31,35,37], RMS Pictorial Scale (RMS-PS) [31], and sound, eye, motor (SEM) scale [36]. Except for one study, according to which there are no differences compared to premedication with anaesthetic gel [36], all the others agree in stating that vibration (and where expected, also cold gel pack) reduces the perception of anxiety and pain related to the injection of the local anaesthetic, also aided by the noise of the vibrational device as a distracting element at the moment of injection.

## 4. Discussion

In this scoping review, we identified nine studies addressing vibrational stimuli as a method aimed at relieving pain during local dental anaesthesia, which were published between 1997 and 2022. Our results indicate a moderate focus of research, in particular, on the dissemination of knowledge and use in the context of the benefit given by local vibration at the time of infiltrative anaesthesia or nerve block. The biological mechanisms underlying this advantageous method in the field of local anaesthesia would be different from those underlying local vibrational stimulation in orthodontics, which are well documented and recognized [22,24,38,39]. From the first study reported in this review, which uses a device with another modified intended use to transfer the vibration at the injection site [32], to the most recent ones mentioned [29,30,31,33,34,35,36,37], in which dedicated devices are used, about twenty years have passed, and undoubtedly also the biological mechanisms to prove its effectiveness are better known. It would seem that “intermittent micro-sonic oscillations to the brain’s neurological pain sensors, closing the pain gate, blocking the pain of injections and is also more useful for paediatric patients and those who have a phobia of intraoral injection or pain as there is an audible distraction (70–75 db) provided” [34]. Stimulation such as vibration can reduce pain based on Ronald Melzack and Patric Wall’s gate control theory. Under this theory of controlling vibration-induced pain relief, painful sensations can be reduced through simultaneous activation of large-diameter nerve fibres that conduct non-harmful stimuli (touch and vibration) [36]. The brain cannot perceive more than one sensation at the same time [32,34,40]. Therefore, the feeling that reaches the brain first will be what the subject will actually feel. So, as a counter-stimulation, vibration reduces painful perception. [41] The oral region is very sensitive because more than a third of the cells in the somatosensory cortex of the brain are dedicated to the sensory inputs of the mouth [32]. The physiological basis of pain relief by vibration is extensively described in one of the most recent studies published on this topic and included in this review. The range of stimulation used stimulates two mechanoreceptors: mainly the Pacinian corpuscle nerve endings, and possibly the Meissner’s corpuscle nerve endings. They are “primarily responsible for vibration detection and high discrimination touch, respectively. Both signals are transmitted via Aβ nerve fibres, which are relatively large-diameter nerve fibres. The pain of injection is transmitted via the small-diameter Aδ and C nerve fibres, primarily Aδ for the pricking pain of needle insertion” and “Vibration or scratching can alter the steady state of the Aβ fibres in a way that exceeds their adaptation potential, which leads to suppression of pain signals transmitting via smaller fibres” [29]. This could explain the efficacy of this method in dental anaesthesia. It would also seem true that the reduction in pain is greater if the source of vibration is applied not only within the area directly affected by the painful stimulus, but also when the application of vibration stimulates the underlying bone on the same side as the perceived pain. This should encourage the development of devices in which the vibratory stimulus is conveyed more and more in a site-specific way with the anaesthetic. The integrated and non-integrated vibration/injection systems currently available are used in comparative studies, always of split mouth type, in order to evaluate how this element is decisive in the effectiveness of the method. As we have seen from the results of this review, the studies reported almost all have a sample of paediatric patients. Although the reason for this choice is not always made clear by the various authors, the reason could lie not only in the fact that children are the most vulnerable category of patients but, as expressed in a study included in the review, the choice of the age range of the sample may be linked to the fact that it is “an age where cognitive development begins to manifest itself” [31]. A final feature that emerged and to consider is that the studies considered almost all refer to both objective and subjective measurements of anxiety and pain. In this review, the studies included made use of five pain measurement scales. The most used scale in the medical field for its simplicity is the VAS (visual analogue scale), especially to describe subjective emotions such as pain [42]. The Iowa Cancer Pain Relief Initiative scale is useful to rate behavioural changes related to pain. Thirty characteristics are assessed, and the participant is asked to rate both the extent to which the behaviour is present and degree of change from baseline [32]. The Wong–Baker FACES pain rating scale is often preferred by parents and patients for reporting pain severity. However, the “no hurt” and “hurts worst” anchor risk to confound pain measurement with non-nociceptive states [43]. The Face, Legs, Activity, Cry, and Consolability (FLACC) scale is one of the most commonly and widely used behavioural observation pain scales [44]. It is also the most frequently used scale used in the studies included in this review [30,31,35,37]. Although developed and validated to evaluate postoperative pain, this scale is currently applied to assess acute pain in multiple settings, including in the emergency department, and it is considered an appropriate observational tool in acute pain assessment in the paediatric population. The RMS Pictorial Scale (RMS-PS) used in one of the studies included in the review [31]. It is an innovative scale for the assessment of a child’s dental anxiety. Also, even if scarcely documented in literature, its application looks promising [45]. One study assessed pain perception using the sound, eye, motor (SEM) scale based on its rating of the patient’s reaction during injection [36]. This scale, such as the FLACC, is often used in combination with other scales to assess the patient’s subjective pain and stress perception, avoiding the use of an operator’s assessment scale [46].

## 5. Conclusions

The use of devices that convey vibrational stimuli to the tissues surrounding those that will be affected shortly thereafter by the injection of anaesthetic, in the different dental procedures with analgesia, can be valuable in terms of reducing the perception of the painful stimulus on the basis of the biological and receptor mechanisms already known. The paediatric indication is currently the most widely documented in literature, but expanding experiences on different age ranges can be important. The operational protocols and the objective and subjective assessment scales to interpret patient responses are still not uniform to provide a reference guide for clinicians who decide they want their patients to benefit from this comfortable operational support.

## Figures and Tables

**Table 1:** Research question based on the PCC (Population/Problem, Concept, Context) strategy.

Population/Problem	Dental Patients
Concept	Application of supplemental vibrational forces decreases pain
Context	Sessions require dental local anaesthesia

**Table 2:** Inclusion and Exclusion criteria.

Inclusion Criteria	Exclusion Criteria
Randomized and non-randomized comparative clinical trials	In vitro and in vivo (animal studies), finite element analysis studies, case reports, case series, reviews
English language	Other languages
Abstract and full text reading available	No abstracts and/or full text reading available

**Table 3:** Research articles included in the scoping review.

Authors and Year	Type of Study	Sample Size and Sex	Age Range	Aim of the Research	Vibrational Device and Protocol Used	Outcomes
Hutchins et al., 1997 [32]	Split mouth Randomized Clinical Trial (RCT)	61 patients; Sex not specified	Adults	To compare the pain perceived during local injections (0.2 mL of 2% lidocaine with 1:100,000 epinephrine administered with a 27-gauge needle penetrating 5 mm of buccal tissue and 2 mm of palatal tissue in the first permanent maxillary premolars areas) with vibration device and topical anaesthetic (20% benzocaine) applied pre-injection.	A battery-powered shaver (Windmere Corp., Miami, FL, USA) was modified to provide the vibration. The blade was removed, and a foam sponge swab was attached. The shaver amplitude was 20μm, and the frequency of vibration was 136 Hz. It was used before the injection.	The measurement of pain perceived by patients was made using a five-point visual analog scale as detailed by the Iowa Cancer Pain Relief Initiative (values 0 to 5). The topical anaesthetic caused a statistically significant decrease in pain values while vibration seems to not improve pain caused by injection.
Nasehi et al., 2015 [33]	Split mouth RCT	99 patients (39 females; 60 males)	Adults	To compare the clinical pain during local anaesthetic injection (lignocaine hydrochloride with adrenaline as vasoconstrictor (1:200,000) (Lox 2%, Neon Laboratories Ltd., Mumbai, India) using or not a vibrational intra-oral device. Injection sites: alveolar inferior, long buccal, infraorbital, palatal.	DentalVibe Injection Comfort system (BING Innovations, Boca Raton, FL, USA). It is a cordless, rechargeable, hand held device that delivers soothing, pulsed, percussive micro-oscillations to the site where an injection is being administered. Manufacturer instructions were applied for its use.	With vibration device, the mean VAS (Visual Analogue Scale) score was significantly lower than without vibration device. This was seen with all the types of local anaesthetic injections.
Shilpapriya et al., 2015 [34]	Split mouth RCT	30 Patients Sex not specified	6–12 years old	To investigate the effects of vibration stimuli on pain experienced during local anaesthetic injections (27 gauge needle) compared to Precaine Gel (Pascal International™) (containing 8% lignocaine and 0.8% dibucaine) applied for 30 s.	DentalVibe Injection Comfort system (BING Innovations, FL, USA) was applied to the injection site 1 min prior to local anaesthesia. The needle was kept as close as possible to one of the prongs. Vibration continued for 10 s after the removal of the needle to help dissipation of anaesthetic solution.	Dental vibe^®^ (Dental Vibe Inc.) is a useful accessory device prior to the use of dental injection syringe and conventional intramuscular injections to alleviate pain and stress of injection. From the aspect of the patient pain management, this device contributes both physiologically (based on Gate Control Theory of pain) and psychologically (based on the audible distraction of the device) and has shown to be a useful tool in patient management.
Alanazi et al., 2019 [35]	Split mouth RCT	60 patients Sex not specified	6–7 years old	To study the discomfort and fear associated with maxillary infiltration injections (1.8 ml of 2% lidocaine with 1:100,000 adrenaline using a 24 mm 30-gauge needle) when using a combination of external cold (gel ice pack comprising of water, sodium polyacrylate and mixed isothiazolinones cooled to 5 °C) and a commercially available vibrating device.	External vibrating device (Buzzy^®^, MMJ labs, Atlanta, GA, USA) was used during the administration of injection.	Children reported a significantly lower Wong–Baker score pain scale and the operators observed a significantly lower heart rate and FLACC (Faces, Legs, Activity, Cry, Consolability) score in the test visit than the control visit. Combining external cold with vibration might be effective in reducing discomfort and fear in children undergoing infiltration dental anaesthesia.
Hedge et al., 2019 [30]	Split mouth RCT	30 patients (15 boys and 15 girls)	6–8 years old (group 1), 9–11 years old (group 2)	To compare the efficacy of a child-friendly device, having a combined effect of vibration and distraction, with the conventional method of injection (including a first step with a topical anaesthetic spray) on pain, anxiety, and behavior of pediatric patients	Not Specified (there is a photo of a fish-shaped device similar to a toy). The children were allowed to touch and turn on the device to familiarize it and not become apprehensive about it. The device was then placed about 2 cm away from the injection site (near the angle of mandible) for 2 min and local anaesthesia was administered.	Results showed that the children (6–11 years old) who received local anaesthesia using the device method had a lower mean pulse rate, FLACC scores, and pain rating scores than those who received local anaesthesia using the conventional method.
Menni et al., 2020 [36]	Split mouth RCT	60 patients (22 boys and 38 girls)	6–12 years old	To evaluate and compare the effectiveness of vibrational device and lignocaine hydrochloride 2% gel (Lox 2% jelly applied for 2 min) in pain reduction during Inferior alveolar nerve block (IANB) for various dental procedures. The IANB was administered using a 2 mL syringe with a 24-gauge needle (Unolok, Hindustan Syringes and Medical Devices Ltd., Faridabad, India) and 2.0 mL of 2% lidocaine with 1:80,000 epinephrine (LIGNOX 2% A, Warren) at a rate of 1 mL/min.	DentalVibe^®^ ([DV] BING Innovations, Boca Raton, FL, USA) was introduced to the injection site with a light touch when contacting the tissue and applied for about 1 min before IANB and continued for 5 s after injecting 2.0 mL of 2% lidocaine.	Both DV and Lox 2% jelly were found to be effective in pain reduction during IANB in children. During both appointments, pain perception was measured using the sound, eye, motor (SEM) scale and Wong–Baker faces pain rating scale (WBFPRS); oxygen saturation (SpO2) and pulse rate were measured using a pulse oximeter before, during, and after the IANB procedure.
Jain et al., 2021 [31]	Split mouth RCT	30 patients Sex not specified	5–10 years old	To evaluate and compare the efficacy of external cold (gel ice pack comprising of water, sodium polyacrylate and mixed isothiazolinones cooled to 5 °C for 30 s) and a vibrating device in reducing the pain and anxiety amidst children receiving maxillary infiltration anaesthesia (1.8 mL of 2% lidocaine with 1:100,000 adrenaline and 26 gauge needle) over conventional methods [2% *w*/*v* lignocaine hydrochloride topical gel for 15–20 s (CALIGNO Jelly, Cachet Pharmaceuticals, Mumbai, India)].	The external vibrating device Buzzy^®^ (MMJ Labs, Atlanta, GA, USA) was applied during anaesthesia injection. Before and after the Buzzy application (and anaesthesia injection) a gel ice pack was applied for 30 s.	Simultaneous to anaesthesia administration, pulse rate and RMS Pictorial Scale (RMS-PS) were employed to measure the child’s discomfort. To document the child’s pain as anticipated by the dentist, the revised face, limbs, arms, cry and consolability (FLACC-R) scale was employed. Lower pain sensation and anxiety was recorded in the experimental group using Buzzy when compared to control. External cold in adjacent with vibrations might be efficient in lowering pain as well as anxiety in children experiencing infiltration dental anaesthesia.
Salma et al., 2021 [29]	Split mouth RCT	166 patients Sex not specified	Not Specified	To evaluate the effectiveness of an electronic hand-held pulsed vibration device on the pain of local analgesia (LA) injection and physiologic changes compared to conventional procedures [prior 2-min application of 2 g topical anaesthesia (benzocaine 20% gel) and 1.8-mL cartridges containing lidocaine 2% with epinephrine 1:100,000 *w*/*v* (0.01 mg/mL)].	DentalVibe Gen 4 (Bing Innovations, LLC, Boca Raton, FL, USA) swtiched on pre- and post-injection.	The vast majority of patients included in our study favoured the use of the DentalVibe over topical anaesthesia. The reduction found in the pain scores when using the device might explain this finding. In addition, the placebo effect and the distraction (which was not measured) caused by the device sound and vibration waves might have influenced the patients’ choice.
Albouni et al., 2022 [37]	Split mouth RCT	75 patients Sex not specified	6–9 years old	To compare the outcomes of the conventional syringe (prior topical gel 20% benzocaine and then lidocaine HCL, 2% and epinephrine 1:100,000) and the outcomes of the vibraject-assisted injection (VAI) in terms of the pain of the needle insertion during various intraoral injections of local anaesthesia (sites of injection: upper posterior buccal, posterior palatal, inferior alveolar nerve block).	Vibraject (Vibraject^®^ MiltexInc LLC., York, PA, USA). It is a battery-operated device that easily clamps on to a syringe and requires little change from conventional injection (CI) techniques. Its protocol of use has not been explained by Authors.	At each clinic visit, subjective and objective pain levels were assessed using the visual analog scale (VAS) and Face, Leg, Activity, Cry, Consolability (FLACC) scale. Children who received local anaesthesia using the Vibraject method had lower VAS and FLACC scores than those who received local anaesthesia using the conventional method. Vibraject was more effective in reducing the pain with local anaesthetic injection compared to the conventional injection technique in clinical dental procedures for children.

## Data Availability

Data will be available on request.
